# Current trends and emerging patterns in the application of nanomaterials for ovarian cancer research: a bibliometric analysis

**DOI:** 10.3389/fphar.2024.1344855

**Published:** 2024-03-08

**Authors:** Wenhui Wang, Jie Wei, Dingqing Feng, Bin Ling

**Affiliations:** ^1^ Department of Obstetrics and Gynecology, China-Japan Friendship Hospital, Beijing, China; ^2^ State Key Laboratory of Chemical Resource Engineering, College of Chemistry, Beijing University of Chemical Technology, Beijing, China

**Keywords:** bibliometric analysis, ovarian cancer, nanomaterial, research trend, visualization

## Abstract

**Introduction:** Ovarian cancer remains to be a significant cause of global cancer-related mortality. In recent years, there has been a surge of studies in investigating the application of nanomaterials in the diagnosis and treatment of ovarian cancer. This study aims to conduct a comprehensive bibliometric analysis regarding nanomaterial-based researches on ovarian cancer to evaluate the current state and emerging patterns in this field.

**Methods:** A thorough literature search on the Web of Science Core Collection database was conducted to identify articles focused on nanomaterial-based ovarian cancer researches. The studies that met the inclusion criteria were selected for further analysis. VOSviewer and CiteSpace were applied for the bibliometric and visual analyses of the selected publications.

**Results:** A total of 2,426 studies were included in this study. The number of annual publications showed a consistent upward trend from 2003 to 2023. Notably, China, the United States, and India have emerged as the leading contributors in this field, accounting for 37.39%, 34.04%, and 5.69% of the publications, respectively. The Chinese Academy of Sciences and Anil K. Sood were identified as the most influential institution and author, respectively. Furthermore, the International Journal of Nanomedicine was the most frequently cited journal. In terms of the research focus, significant attention has been directed towards nanomaterial-related drug delivery, while the exploration of immunogenic cell death and metal-organic frameworks represented recent areas of interest.

**Conclusion:** Through comprehensive analyses, an overview of current research trends and emerging areas of interest regarding the application of nanomaterials in ovarian cancer was illustrated. These findings offered valuable insights into the status and future directions of this dynamic field.

## 1 Introduction

Based on global estimates, approximately 314,000 cases of ovarian cancer (OC) are diagnosed annually, with around 207,000 cases of death each year (Sung et al., 2021). OC is the primary cause of death among gynecologic cancers in the United States and ranks as the fifth most prevalent cause of cancer-related mortality in women (Armstrong et al., 2021). The predominant challenges in managing OC lie in the advanced stage of the disease in initial diagnosis and the lack of effective corresponding therapeutic strategies (Armstrong et al., 2021; Khan et al., 2021). Despite the utilization of various treatment schemes, such as combining biological agents with chemotherapies to impede tumor growth and minimize recurrence, the limited bioavailability of the drugs and their non-specific activation yield diminish the therapeutic efficacy and cause severe side effects to patients (Lustberg et al., 2023). Consequently, there is an urgent necessity for innovative and efficient methods to diagnose and treat OC.

The development of nanotechnology provides a groundbreaking platform for novel material-based diagnosis and imaging of diseases with enhanced efficacy and properties, due to their distinct characteristics including adjustable size, strong affinity, stability, labeling function, thermal properties, and internalization capacity (Rajitha et al., 2021). The integration of nanotechnology and pharmaceutical sciences has triggered a revolution in the medical domain. Numerous nanomaterials have been approved by the United States Food and Drug Administration (FDA) for the application in anticancer medications and diagnostic agents, and numerous clinical trials have been conducted to examine their potentials (Nirmala et al., 2023).

Nanotechnology thus provides novel molecular agents that could enable OC diagnosis at initial stages and allow continuous monitoring during treatment (Rajitha et al., 2021). By serving as contrast agents, molecular imaging agents and intraoperative aids, novel nanomaterials improve traditional clinical methods by recognizing OC early and precisely positioning it (Henderson et al., 2021). For instance, Williams et al. reported a optically responsive carbon nanotube to detect the OC biomarker HE4 *in vivo* (Williams et al., 2018). Pu T et al. developed nanoparticles with near-infrared-II fluorescence (NIR-II NPs) can accurately detect early orthotopic and advanced-stage metastatic OC in mice models (Pu et al., 2023). Additionally, the utilization of nanoparticles (NPs) can also facilitate localized drug delivery, enhance drug retention, and minimize systemic toxicity when treating OC (Bhattacharya et al., 2022; Zhang et al., 2023). Examples of NP formulations that have received FDA approval for the treatment of OC include Doxil^®^, which is a liposomal formulation of doxorubicin and Abraxane^®^, which is a human serum albumin nanoaggregate of paclitaxel (Barenholz, 2012; Li et al., 2020; National Comprehensive Cancer Network, 2023).

Nevertheless, most of the nanomaterial products in nanomedicine are still in the stage of *in vitro* cell culture or *in vivo* animal experiments. There are several possible reasons why nanostructures have not improved clinical practice in OC as expected. Regulatory issues, safety concerns, nanomedicines’ physicochemical characteristics and manufacturing problems may account for this (Zhang et al., 2023). Consequently, it is promising to use nanomaterials in cancer diagnosis and treatment, but many challenges must be overcome before they can be used clinically.

Bibliometric analysis is a statistical approach that utilizes public literature databases to conduct quantitative and qualitative evaluation on publications of interest, by which the research trends and hotpots within a specific field can be concluded. Recently, bibliometrics has been employed in the investigation of nanomaterials (Bhandari et al., 2022; Zhao et al., 2022; Han et al., 2023). However, there is a dearth of bibliometric analysis regarding the utilization of nanomaterials in the context of OC. Therefore, this study utilized a quantitative methodology to illustrate the current situation of nanomaterials applied in OC treatment. Therefore, such quantitative methodology was utilized in this study in order to illustrate the current situation of nanomaterials applied in OC treatment. In this study, based on the Web of Science Core Collection (WOSCC) database, we used software such as VOSviewer, CiteSpace, and Pajek to conduct bibliometric and visual analysis on the research trends of countries/regions, institutions, authors, publications, citations, and keywords in the nanomaterials and OC field. This analysis helped identify research hotspots and provides suggestions for future research directions.

## 2 Methods

### 2.1 Data acquisition and filtration

**FIGURE 1:**
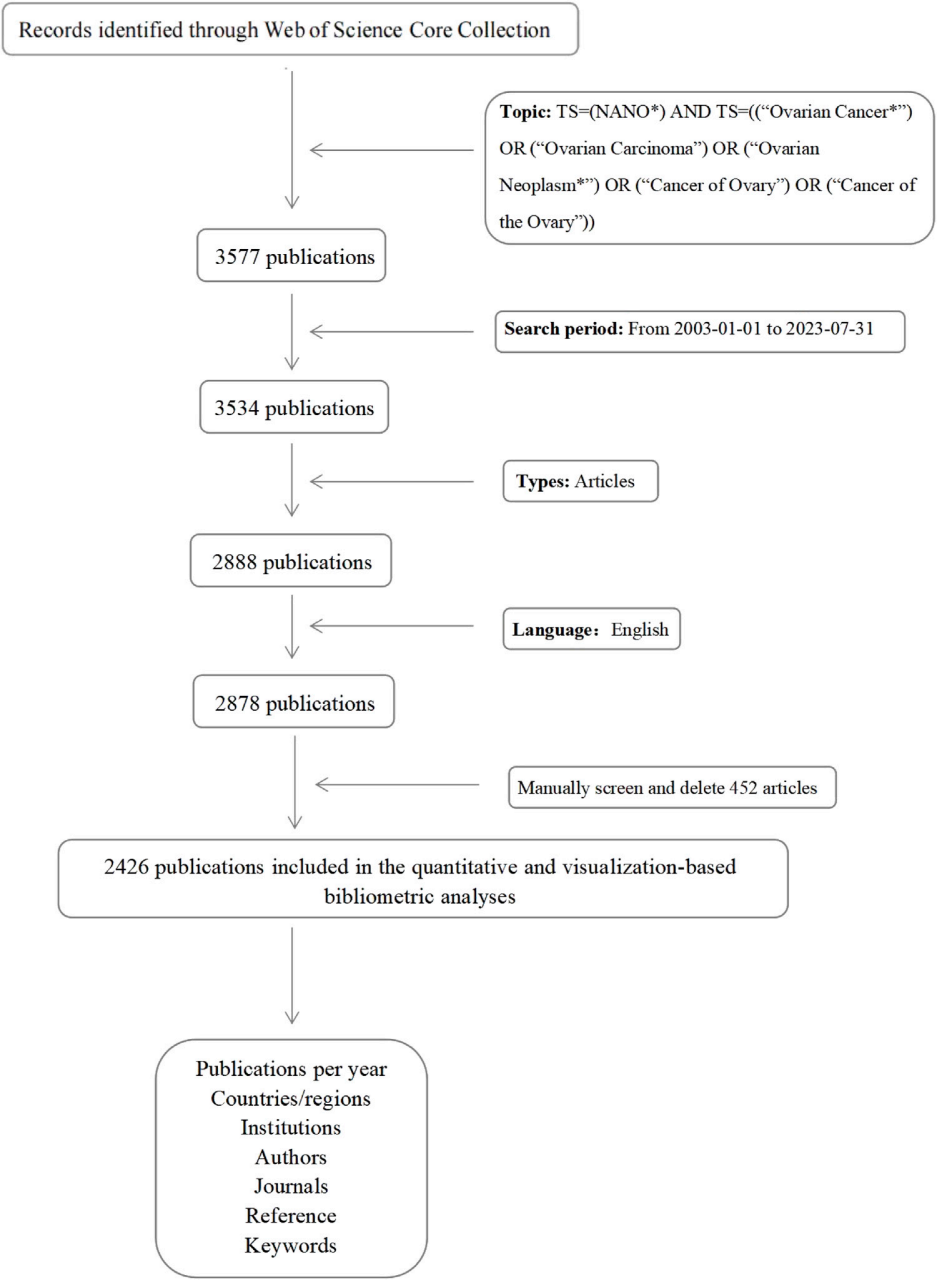
Flowchart of the literature screening process.

To conduct data retrieval, we utilized the WOSCC database on 31 July 2023. The employed search formula was TS=(NANO*) AND TS=((“Ovarian Cancer*”) OR (“Ovarian Carcinoma”) OR (“Ovarian Neoplasm*”) OR (“Cancer of Ovary”) OR (“Cancer of the Ovary”)). The inclusion of the term “nano*” allowed the search to encompass all the terms beginning with “nano”, such as nanoparticles, nanomaterials, nanocomposites, nanocarriers, nanotechnology, and so on. The timeframe of the search spanned from 2003 to 2023, constituting a 20-year period. During the initial screening stage, only articles were included, while the irrelevant documents such as reviews, meeting abstracts, biographical-items, editorial materials, early access articles, letters, book chapters, proceeding papers, corrections, news items, and retracted papers were excluded. Furthermore, in order to refine our analysis, articles with reported contents unrelated to nanomaterials in the context of OC were manually excluded (Figure 1).

### 2.2 Data analysis

In our present investigation, VOSviewer (v1.6.18), CiteSpace (v6.1.6), Pajek (v5.16), Scimago Graphica (v1.0.35), and R-bibliometrix (v4.1.0) were applied to perform bibliometric and visual analyses. VOSviewer was mainly responsible for generating visual graphs and examining the countries, institutions, and authors with the most prolific collaborations, as well as the most frequently cited journals and cooccurring keywords. Meanwhile, CiteSpace was employed to construct a timeline graph and identify the bursts of keyword terms. Each dot on the visual graphs corresponds to a country, institution, author, or journal, and these dots were grouped based on their collaborative efforts. The size of the dot was dependent on the number of publications. Link strength (LS) was the thickness of the line connecting the nodes and represented the strength of cooperation between them, and total link strength (TLS) reflected the overall level of cooperation. In the keyword analysis, several insignificant keywords were excluded, and those with similar meanings were merged to gain a better perspective.

## 3 Results

### 3.1 Analysis of general trend

**FIGURE 2:**
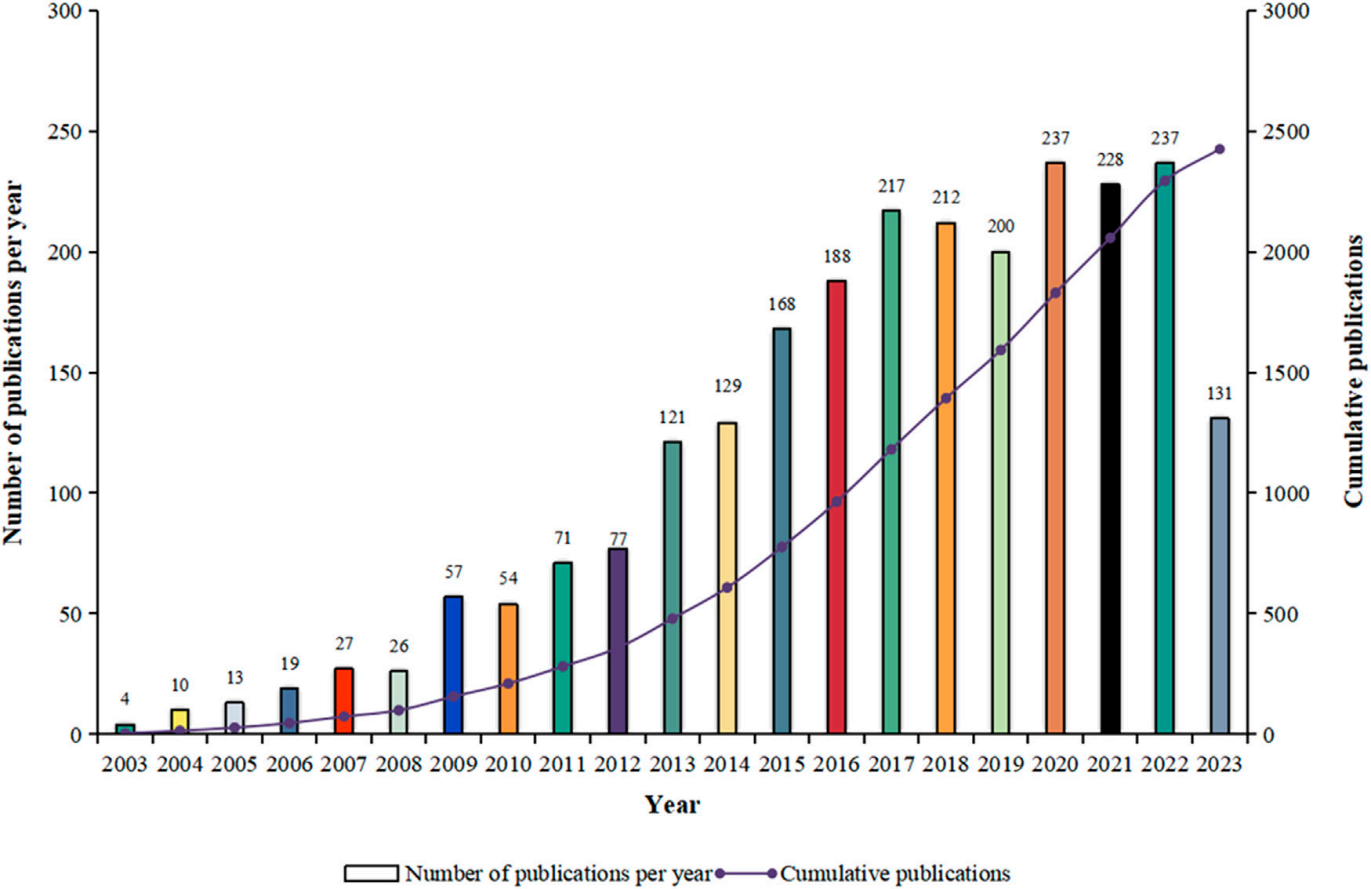
Trends in the volume of publications per year of nanomaterials in ovarian cancer.

In this study, a total of 2,426 related documents were identified and met the inclusion criteria, and the annual scientific productions showed a general ascending trend, indicating that attention to the field of OC and nanomaterials increased (Figure 2).

### 3.2 Analysis of countries/regions

**FIGURE 3:**
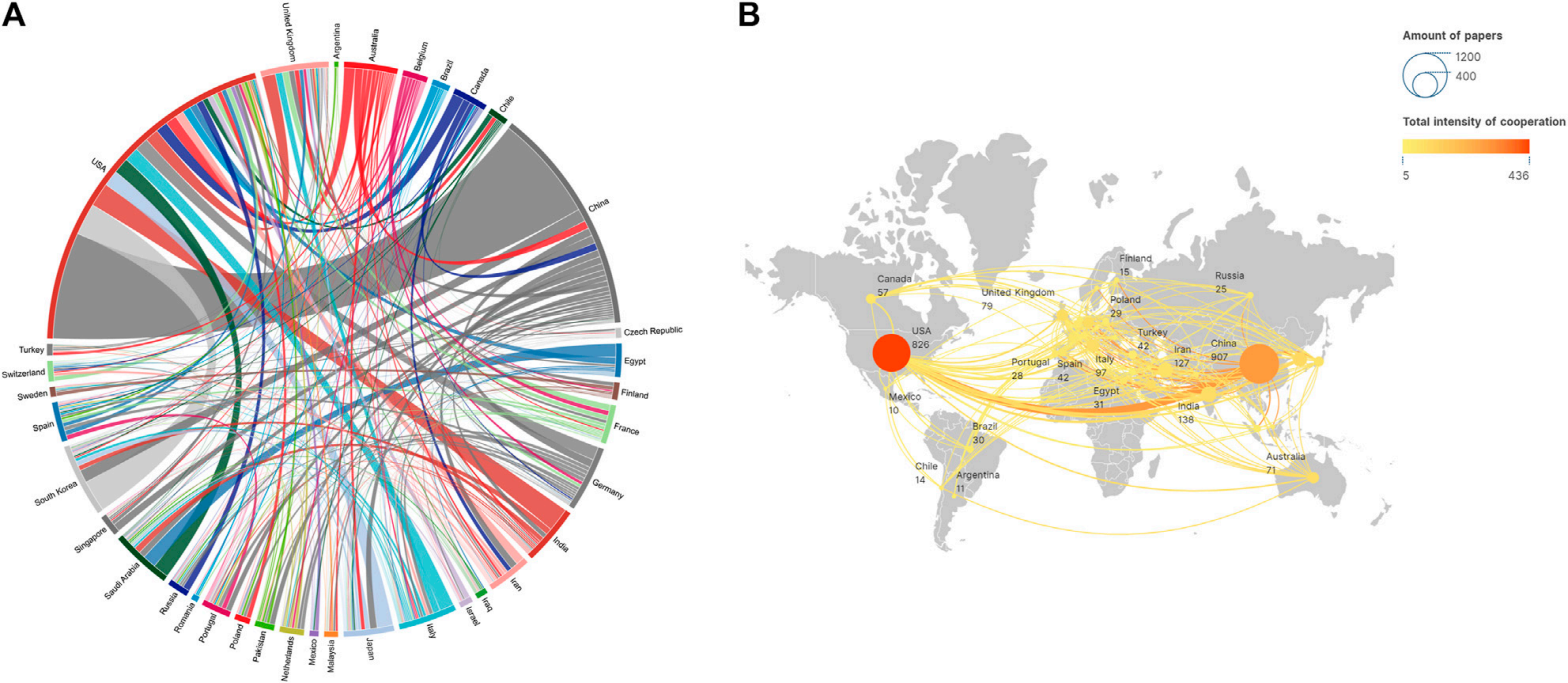
**(A, B)** The coauthorship network map of countries.

The coauthorship network visualization map of countries is shown in Figures 3A,B. A total of 72 countries/regions were presented. The United States exhibited the strongest international collaboration network (TLS = 436), which had the closest cooperation with China (LS = 134). Next, the number of publications was analyzed, revealing that China had the highest publication count (907, 37.39%), followed by the United States (826, 34.04%), and India (138, 5.69%).

### 3.3 Analysis of institutions

**FIGURE 4:**
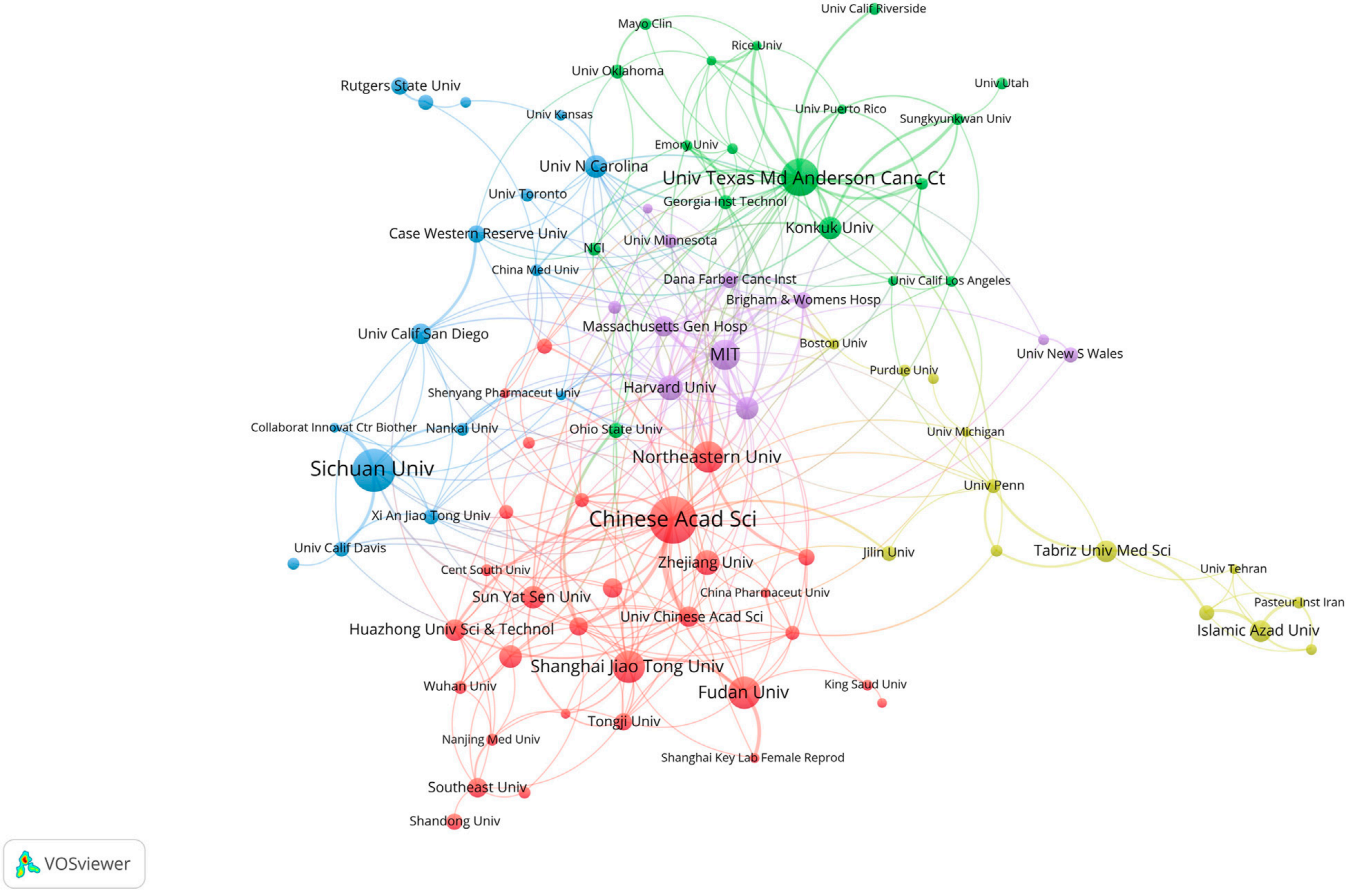
The coauthorship network map of institutions.

**FIGURE 5:**
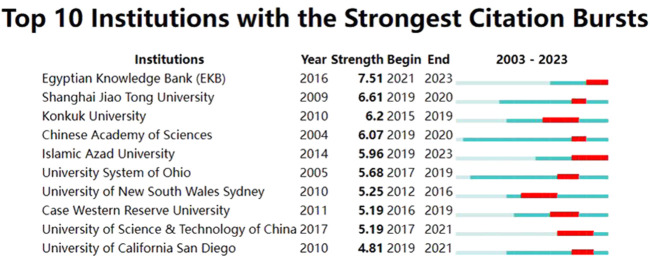
Top 10 institutions with the strongest citation bursts.

From 2003 to 2022, a total of 2,438 institutions conducted studies in this field. The top three institutions were the Chinese Academy of Sciences (85 publications), Sichuan University (75 publications), and The University of Texas MD Anderson Cancer Center (62 publications). Institutions that had at least 10 publications were included in the analysis of collaborative networks which were visualized by VOSviewer. The clusters were arranged in different colors based on the frequency of collaboration between institutions (Figure 4). The Chinese Academy of Sciences had the largest node (TLS = 92), indicating the highest level of collaboration with other institutions. The strongest connection was between the Chinese Academy of Sciences and the University of Chinese Academy of Sciences (LS = 32), which was represented by the thickest line. Figure 5 depicted the publications of the top 10 institutions with the most significant citation bursts, as indicated by the red bars. The publications of the Egyptian Knowledge Bank (EKB) and Islamic Azad University experienced a sharp increase from 2021 to 2023, with a burst intensity of 7.51 and 5.96, suggesting an increasing focus on the researches related to OC and nanomaterials during the past 3 years.

### 3.4 Analysis of authors

**FIGURE 6:**
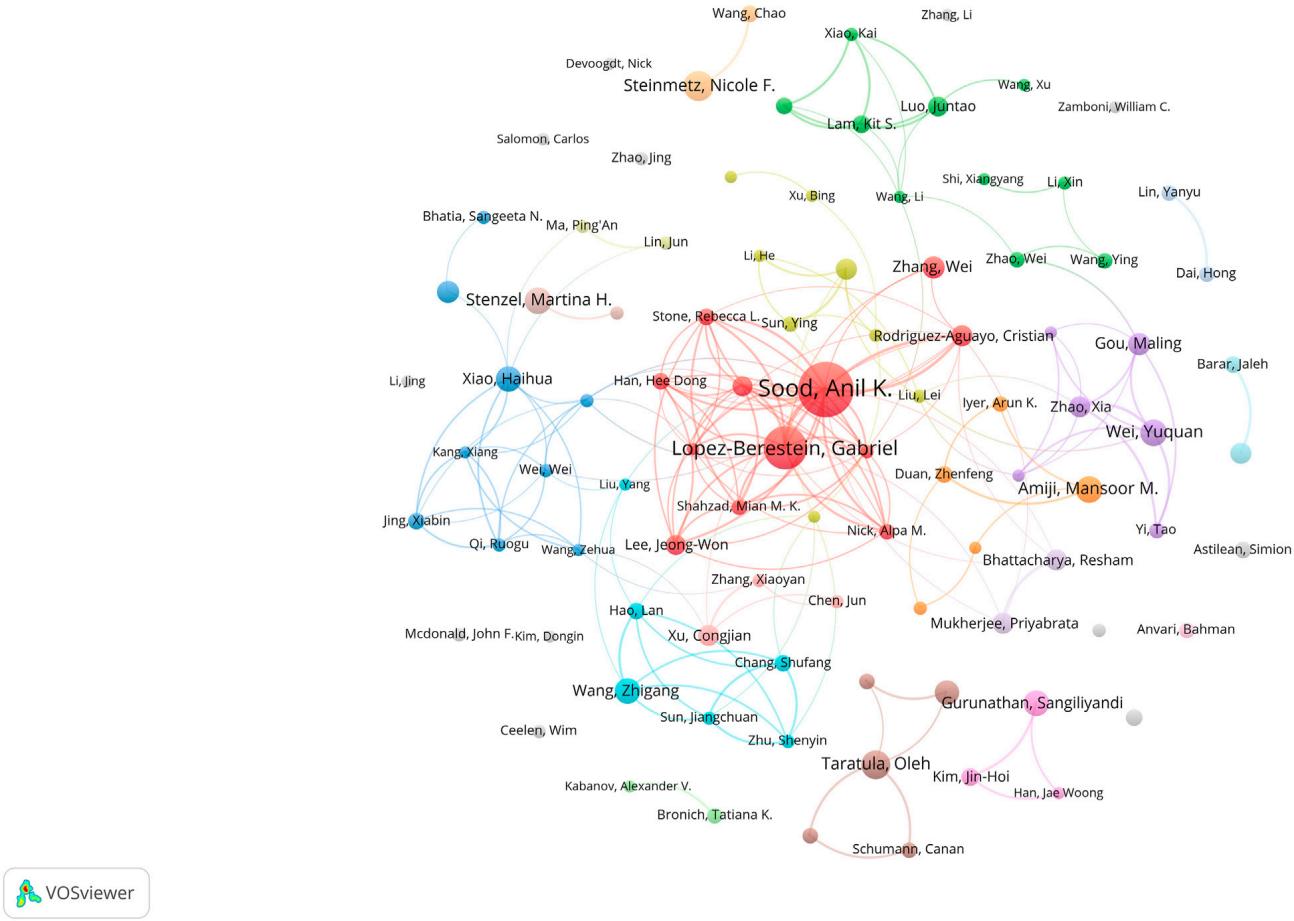
The coauthorship network map of authors.

**FIGURE 7:**
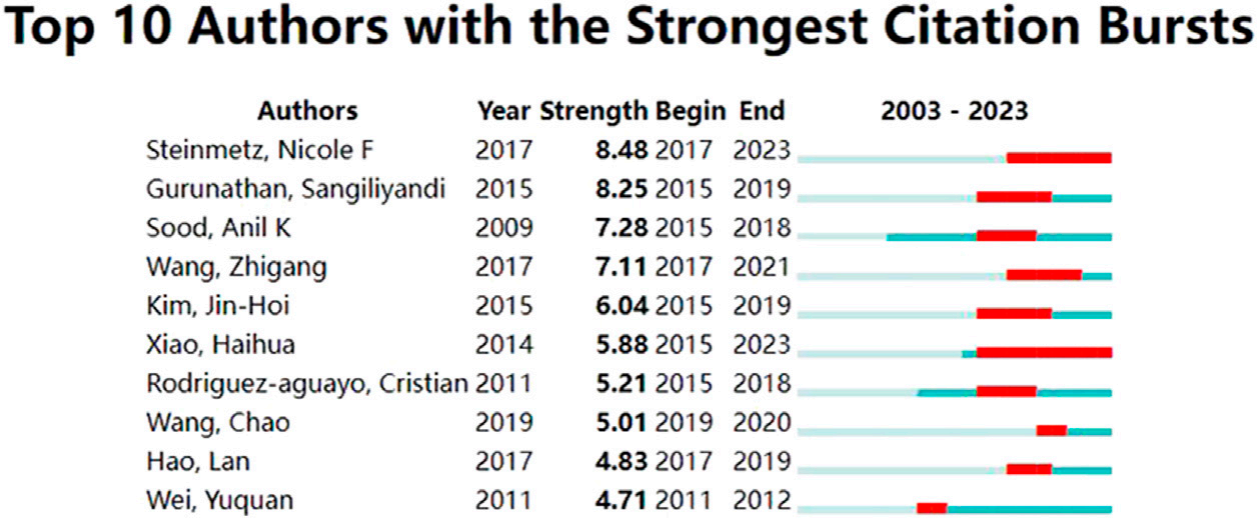
Top 10 authors with the strongest citation bursts.

The author collaboration network map was presented in Figure 6. A total of 13,140 authors have published relevant papers, with Anil K. Sood being the most influential author with 38 publications, followed by Gabriel Lopez-Berestein and Nicole F. Steinmetz. Anil K. Sood gained the highest number of collaborative relationships with other authors. Anil K. Sood and Gabriel Lopez-Berestein from the University of Texas MD Anderson Cancer Center in the United States possessed the closest collaboration. Figure 7 illustrated the citation bursts of the top ten authors, with the time interval and duration of the bursts marked in blue and red, respectively. Steinmetz, Nicole F from the University of California in the United States, as well as Xiao Haihua from the Chinese Academy of Science in China, have experienced a significant increase in their publication output in the past 3 years. This indicated a notable surge of their creativity in the nanomaterials and OC field.

### 3.5 Analysis of journals

**FIGURE 8:**
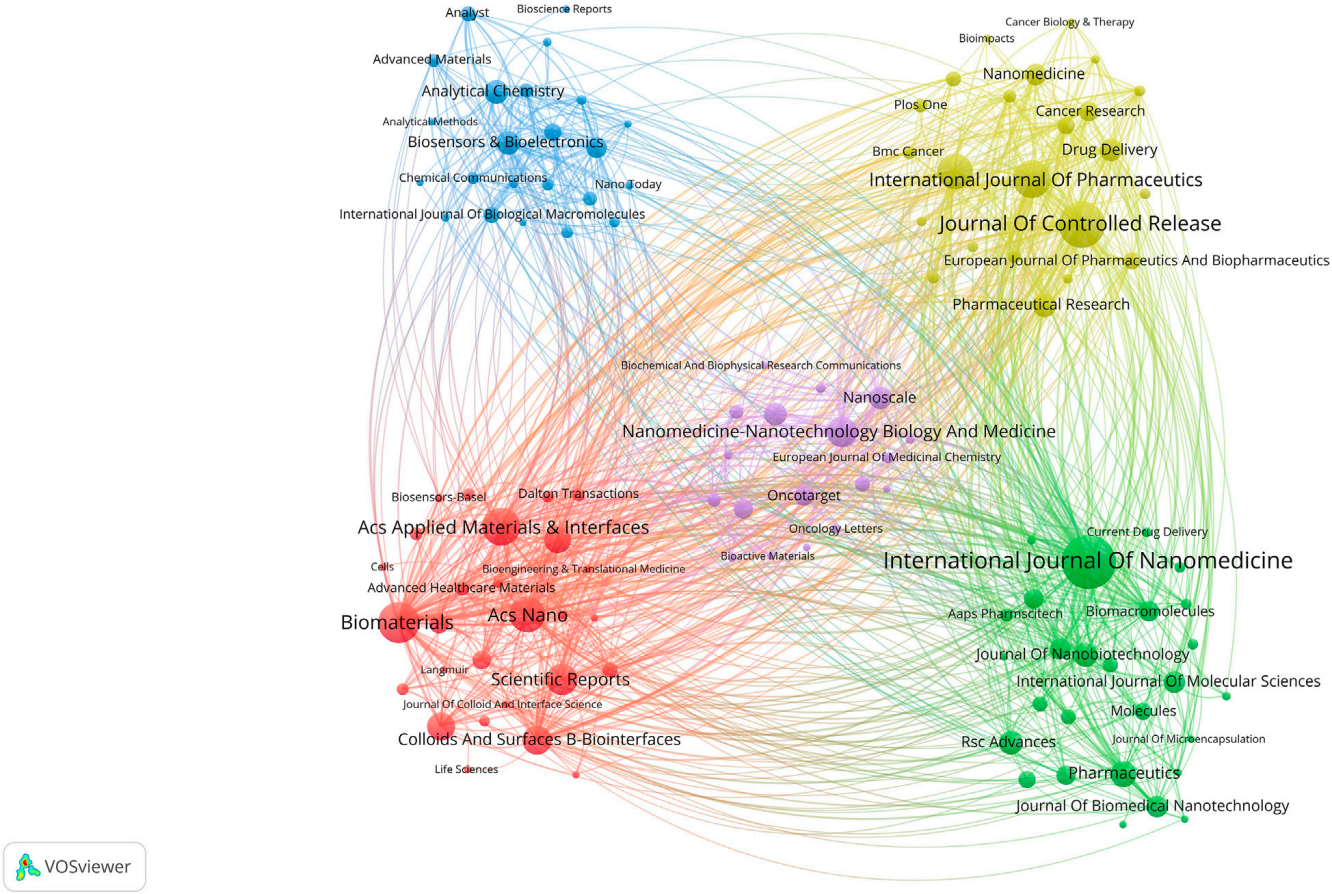
The cocitation network map of journals.

Since 2000, a total of 558 journals have published articles related to nanomaterials and OC, as shown in Table 1. Among those journals, ACS Nano has the highest impact factor (IF 2022 = 17.1), and 85% of the journals reached a JCR partition of Q1. We further refined our analysis by filtering out 118 journals with fewer than 5 relevant publications, resulting in 5 distinct clusters as presented in Figure 8. Larger nodes in the figure indicated a greater number of relevant publications, while the connecting lines between nodes represented cross-citation relationships between two journals. It was notable that the journals publishing research on nanomaterials in the field of OC demonstrated an active citation relationship.

**TABLE 1:** Top 10 journals in terms of the number of published papers.

Rank	Journal	Record	Country	IF(JCR 2022)	JCR quatile
1	International Journal of Nanomedicine	85	New Zealand	8.0	Q1
2	Journal Of Controlled Release	68	Netherlands	10.8	Q1
3	Biomaterials	56	Netherlands	14	Q1
4	Acs Applied Materials and Interfaces	49	United States	9.5	Q1
5	International Journal Of Pharmaceutics	49	Netherlands	5.8	Q1
6	Acs Nano	45	United States	17.1	Q1
7	Molecular Pharmaceutics	45	United States	4.9	Q1
8	Scientific Reports	38	England	4.6	Q1
9	Nanomedicine-Nanotechnology Biology And Medicine	36	Netherlands	5.4	Q2
10	Colloids And Surfaces B-Biointerfaces	34	Netherlands	5.8	Q1
11	Journal Of Materials Chemistry B	32	England	7.0	Q1
12	Bioconjugate Chemistry	29	United States	4.7	Q1
13	Pharmaceutics	28	Switzerland	5.4	Q1
14	Analytical Chemistry	24	United States	7.4	Q1
15	Biosensors and Bioelectronics	24	Netherlands	12.6	Q1
16	Journal Of Nanobiotechnology	24	England	10.2	Q1
17	Pharmaceutical Research	24	Germany	3.7	Q2
18	Rsc Advances	24	England	3.9	Q1
19	Cancers	23	Switzerland	5.2	Q2
20	Drug Delivery	23	United States	6.0	Q1

**FIGURE 9:**
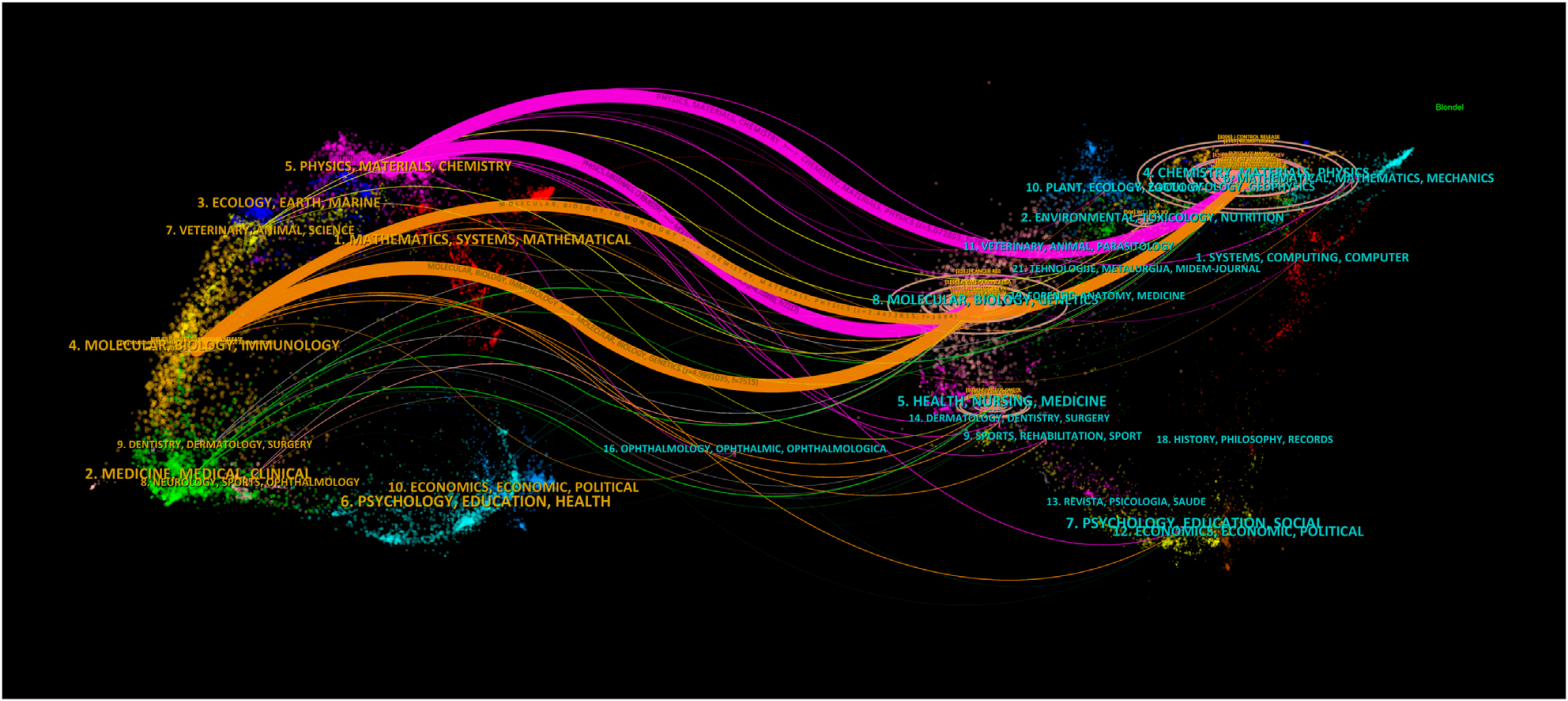
The dual-map overlay of journals contributed to publications.

Figure 9 illustrated the dual-map overlay of journals contributing to publications in the field of nanomaterials and OC from 2003 to 2023. The left side represented the citing journals, while the right side represented the cited journals. The colored line paths indicated the citation relationships. The analysis revealed that the research primarily focused on journals in the fields of physics, materials, chemistry, molecular biology, immunology, medicine, and clinical studies. The cited journals were predominantly from the fields of molecular biology, genetics, chemistry, materials, and physics. This interdisciplinary network and collaboration actively reflects current trends in multiple fields, especially at the forefront of nanomaterials and medicine.

### 3.6 Analysis of co-cited references

**FIGURE 10:**
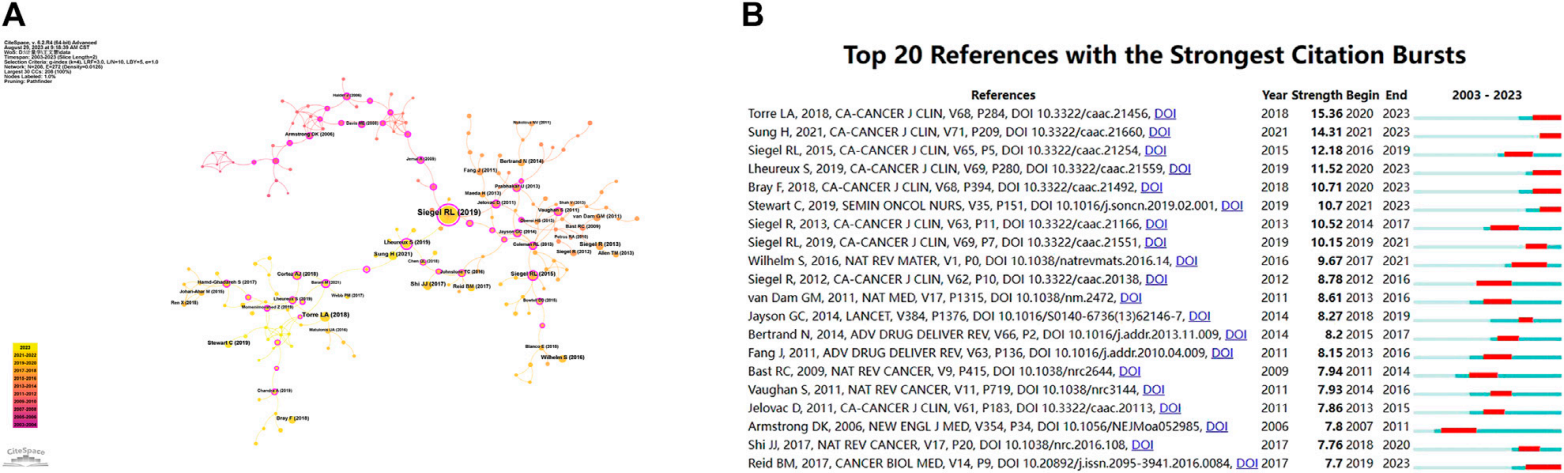
**(A)** Labels clustering of co-cited literature and **(B)** the top 20 references with the strongest citation bursts.

Table 2 presented the top 10 co-cited publications related to nanomaterials and OC. The visualization of the co-cited publications was realized by CiteSpace software, with larger labels assigned to authors based on the number of citations (Figure 10A). It was noteworthy that the publication by Professor Siegel RL from the United States, which focused on the annual estimates and the latest data on cancer incidence, mortality, and survival in the United States in 2018, received the highest citations, of 152 (Siegel et al., 2019). This publication was highly valuable for epidemiological studies since it provided the annual estimates of new cancer cases and deaths in the United States, along with the most recent data on population-based cancer incidence. Among the top 10 co-citations, the journal “CA: A Cancer Journal for Clinicians” holds the highest impact factor (IF 2022 = 254.7), followed by “Nature Reviews Cancer” (IF 2022 = 78.5). Furthermore, we utilized CiteSpace to identify the top 20 references that experienced a strong citation burst. A citation burst refers to references that have garnered substantial attention from other studies over a specific time period. Figure 10B illustrated that, since 2003, the strongest citation burst originated from the paper by Torre et al. (Torre et al., 2018)in 2018, followed by the article by Sung et al. (Sung et al., 2021) on CA-CANCER J CLIN in 2021 and the article by Lheureux et al. (Lheureux et al., 2019) on CA-CANCER J CLIN in 2019.

**TABLE 2:** Top 10 cited publications.

Rank	Co-cited references	Total citations	Centrality	Journal	IF(2022 years)	Corresponding author’s country
1	CA: A Cancer Journal for Clinicians	152	1	CA-CANCER J CLIN	Q1/254.7	United States
2	Ovarian cancer statistics, 2018	44	0.08	CA-CANCER J CLIN	Q1/254.7	United States
3	CA: A Cancer Journal for Clinicians	32	0.25	CA-CANCER J CLIN	Q1/254.7	United States
4	CA: a cancer journal for clinicians	32	0.02	CA-CANCER J CLIN	Q1/254.7	United States
5	Cancer nanomedicine: progress, challenges and opportunities	29	0	NAT REV CANCER	Q1/78.5	England
6	CA: A Cancer Journal for Clinicians	28	0.02	CA-CANCER J CLIN	Q1/254.7	United States
7	Epithelial ovarian cancer: Evolution of management in the era of precision medicine	27	0.76	CA-CANCER J CLIN	Q1/286.130	United States
8	Analysis of nanoparticle delivery to tumours	26	0	NAT REV MATER	Q1/83.5	England
9	Ovarian Cancer: An Integrated Review	24	0	SEMIN ONCOL NURS	Q2/2.2	United States
10	Cancer nanotechnology: The impact of passive and active targeting in the era of modern cancer biology	22	0.05	ADV DRUG DELIVER REV	Q1/16.1	Netherlands

### 3.7 Analysis of keywords

Keyword co-occurrence analysis was conducted to identify popular research topics. Supplementary Figure S11A,B showed the network and overlay visualization maps of co-occurring keywords. The top 10 frequent keywords were OC, drug delivery, NPs, paclitaxel, cisplatin, doxorubicin, folic acid, apoptosis, chemotherapy, and nanomedicine. These keywords represented the key research areas within the field of nanomaterials and OC. The cluster analysis of the network map accurately reflected the knowledge structure of the research fields. By using VOSviewer software, we conducted a co-occurrence clustering analysis and visualization of keywords in the literature. A total of 132 out of 4,842 keywords were analyzed to achieve a minimum occurrence frequency of 7. Among these, the top 10 frequent keywords were OC, drug-delivery, NPs, paclitaxel, cisplatin, doxorubicin, cancer, folic acid, apoptosis, and chemotherapy. In Supplementary Figure S11A, the network map displayed 6 distinct clusters which were represented by different colors. The largest cluster marked in red was focused on the NP-based diagnosis and treatments, with prominent keywords such as “CA125”, “biomarkers”, “biosensor”, and “gene therapy”. The second largest cluster marked in green was mainly associated with nanotechnology, and keywords like “liposomes”, “nanocarriers”, “gene delivery”, and “self-assembly” were included. The blue marker, representing the third largest cluster, committed to drug delivery and loading agents, with keywords like “paclitaxel”, “cisplatin”, “doxorubicin”, and “siRNA” being central to this cluster. The yellow cluster was related to the targeting and imaging, with significant keywords such as “folic acid”, “EGFR”, “MRI”, and “magnetic NPs”. The purple cluster which was focused on targeting and therapy was characterized with keywords such as “cancer stem cell”, “ph-sensitive”, “photodynamic therapy”, and “photothermal therapy” being prominent. Lastly, the light blue cluster explored the anticancer mechanisms with frequent keywords such as “apoptosis”, “cytotoxicity”, “reactive oxygen species”, and “autophagy”.

The trend topic analysis was an important mapping tool that helped to portray the seed of trend integration rooted in the previous stream (Zhao et al., 2022). Supplementary Figure S11B, terms marked in purple indicate that their average year of publication was 2015 or earlier, while those marked in bright yellow appeared after 2019. Keywords such as “proteomics” and “nanoemulsion” were the main topics during the early stage. The keywords “immunogenic cell death” and “metal-organic framework” appeared relatively late in the study period.

**FIGURE 11:**
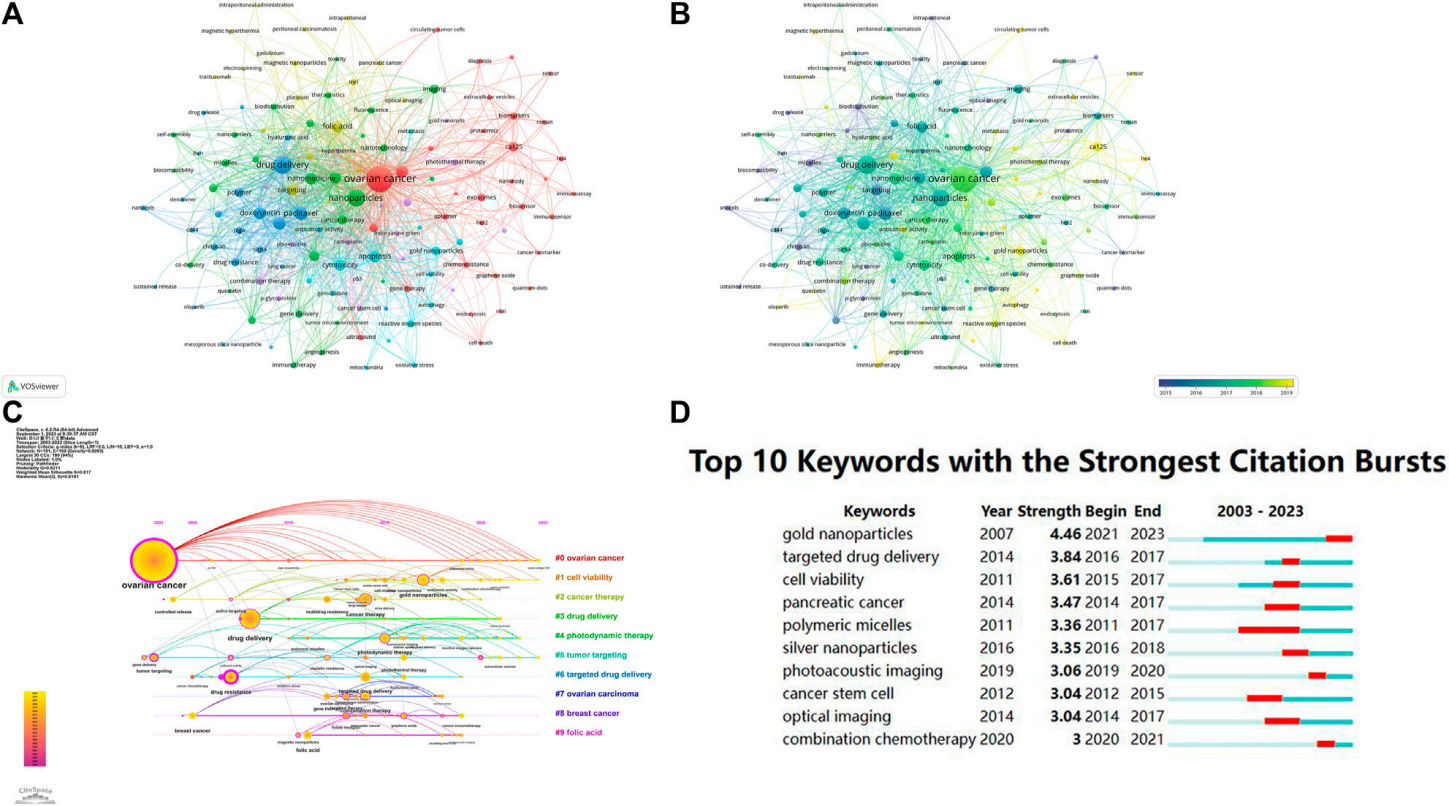
The network **(A)**, overlay **(B)**, and timeline **(C)** map of keyword co-occurrence. **(D)** the top 10 keywords with the strongest citation bursts.

In addition, we presented a visualization of the keyword evolution over time using CiteSpace (Figure 11C). Before 2010, the main hot research keywords were OC and drug delivery. As of 2023, OC, cell viability, and tumor targeting continue to be hot topics. Another important indicator of the study frontiers and hotspots over time was the strength of the keyword bursts (Figure 11D). Among the top 10 keywords with the strongest citation bursts, gold NPs had the highest burst strength in the last 3 years, suggesting that they are still popular research subjects.

## 4 Discussion

To our knowledge, for the first time this study conducted a comprehensive bibliometric analysis on the application of nanomaterials in OC between 2003 and 2023, which provided an overview of the global research landscape in this field, identified the research hotspots, and made predictions about future trends. According to the results, the research in this area has been developing rapidly over the past 2 decades.

Notably, China and the United States have emerged as the main contributors in this field. China has the highest number of publications, while the United States achieves the highest citation frequency and has the most extensive collaborations with other countries or regions. Among the top institutions in publication output, the Chinese Academy of Sciences stands out as the most productive institution in the collaborative network, with the highest overall link strength. In terms of publication output, The International Journal of Nanomedicine ranks as the top in this field. According to the authors’ viewpoint, KSood, Anil K turn to be the most influential authors, closely followed by Lopez-Berestein Gabriel, who are both affiliated with the University of Texas MD Anderson Cancer Center in the United States. Notably, Steinmetz, Nicole F from the University of California in the United States and Xiao, Haihua from the Chinese Academy of Science have made significant contributions with extensive publication records in the past 3 years. These noteworthy achievements could be attributed to the support provided by institutions in both China and the United States, including their policies and financial funding, which have fostered extensive and in-depth researches in this particular field.

The keyword analysis revealed that the most frequently recurring keywords were associated with drug delivery, emphasizing its status as an extensively studied subfield. Notably, recent hot spots in the research keywords included “immunogenic cell death” and “metal-organic framework,” indicating the latest areas of interest in this field. Among these hot spots, the researches on gold NPs have obtained remarkable burst strength over the last 3 years, holding promising potentials in OC diagnosis, treatment, and drug delivery, both *in vivo* and in vitros. Furthermore, recent studies have shed light on the significant role of nanomaterials in cancer immune regulation. With proper optimization in the structure and functions, nanoparticles can be utilized to directly reverse the immune status of primary tumors, stimulate the potential of peripheral immune cells, prevent the formation of pre-metastatic niches, and suppress tumor recurrence through postoperative immunotherapy (Zhang et al., 2021; Li et al., 2022). Therefore, the combination of advanced immunotherapy and novel nanomaterials is burgeoning as a reliable scheme for the treatment of refractory and metastatic malignancies in the future.

The unique physical and chemical properties of nanomaterials facilitate a wide rage of applications, especially in the field of cancer therapy. OC is a highly fatal gynecological malignancy worldwide, which is known for its significant morbidity and mortality. Conventional chemotherapeutic drugs often fail to achieve satisfying effects in treating OC due to drug resistance. However, the auxiliary application of nanomaterials can enhance drug accumulation in tumors, reduce off-target toxicity, prevent rapid drug clearance after systemic delivery, and improve the pharmacokinetics of the drug, ultimately leading to higher therapeutic efficiency (Baranello et al., 2014; Chen et al., 2021). Nano-technique is considered as an effective approach to address the poor aqueous solubility of hydrophobic drugs. For example, exosomal and liposomal nano-carriers for mangiferin and curcumin exhibited increased cellular uptake and controlled release (Alharbi et al., 2024). Nanocarriers have also been used to specifically target to tumors, such as the tumor-targeted probes for Follicle-stimulating hormone (Liu et al., 2024). Furthermore, multifunctional nanoparticles (DDP-Ola@HR) which were loaded with both DDP and Olaparib and modified with heparin, have shown effectiveness in inhibiting the growth and metastasis of DDP-resistant OC(Liang et al., 2023). However, there are challenges that need to be addressed. Long-term toxicity and side effects of nanomaterials require further study, since the verification of bio-safety on the monthly bases is too short to fully understand. In addition, the complex manufacturing process of nanomedicines can result in inconsistency between different batches, and for the quality control of laboratory manufacture, there is still a long way to reach the criteria of clinical application.

Despite these challenges, nanomaterials hold the promise in various treatment approaches for OC, including phototherapy, chemotherapy, targeted therapy, and combination therapy. The limitations of chemotherapeutic drugs, such as poor water solubility, adverse side effects, and multidrug resistance, can be removed with the use of nanomaterials. In recent years, there have been developments in the use of MO based NPs to address solubility issues of hydrophobic drugs. The drug was loaded in nanocarriers through encapsulation, with a high drug loading rate up to 10 wt% (Zhai et al., 2018). Another approach focused on the use of nano-micelles to deliver anti-tumor drugs such as betulinic acid and paclitaxel, which has shown promising therapeutic efficacy in tumors with multidrug resistance (Qu et al., 2023). To overcome the limitations of monotherapy, a multi-mode nanoplatform called Fe-Dox/PVP was designed to combine chemotherapy, ferroptosis, and mild photothermal therapy. This nanoplatform exhibited significant anticancer effects both *in vitro* and *in vivo* (Dai et al., 2023). Overall, more researches are needed in order to fully address the problem of OC and make the most of the advantages nanomaterials.

The application of nanotechnology in treating OC has experienced significant growth over the past 2 decades. A number of clinical trials have been conducted to assess the effectiveness of nanomedicines in treating relapsed or refractory OC. These studies have utilized nanocarriers to load various drugs, including doxorubicin (NCT0148937), paclitaxel (NCT02125662), cisplatin (NCT02790858), oxaliplatin (NCT02565349), carboplatin (NCT03071672), and siRNA (NCT02541521). The results of these trials verified the potential and feasibility of nanomedicines in treating OC. Notably, the FDA has approved Doxil^®^ (liposomal doxorubicin), and Abraxane^®^ (albumin-bound paclitaxel) as two important chemotherapy options for the clinical treatment of OC (Barenholz, 2012; Li et al., 2020; National Comprehensive Cancer Network, 2023). In recent years, researchers have been drawing growing attention on nanomedicine carrier design, precisely targeted drugs, and multimodal combined multimodal therapy (Li et al., 2020). Overall, improvements in clinical research on OC therapy have achieved, although further research and exploration are still necessary to develop strategies with better therapeutic effects and higher bio-safety.

It is undeniable that there are limitations in this study. The literature search was limited to the WoSCC database, which may have resulted in an incomplete conclusion. nevertheless, the WoSCC database is widely recognized as one of the most comprehensive sources for bibliometric analysis. Additionally, only studies published in English were included.

In conclusion, this study utilized various statistical software programs to conduct a bibliometric analysis on nanomaterials in the diagnosis and treatment of OC. The advantages and challenges faced by nanomaterials in this field were discussed. Nanomaterials have the potential to be a powerful tool in the diagnosis and treatment of OC. This study provides valuable insights into the recent developments and trends in the use of nanomaterials for treating OC, offering researchers a conclusive and convenient port to learn about the current circumstances in this field National Comprehensive Cancer Network, 2023.

## Data Availability

The original contributions presented in the study are included in the article/Supplementary Material, further inquiries can be directed to the corresponding authors.

## References

[B1] Alharbi H. M. Alqahtani T. Alamri A. H. Kumarasamy V. Subramaniyan V. Babu K. S. (2024). Nanotechnological synergy of mangiferin and curcumin in modulating PI3K/Akt/mTOR pathway: a novel front in ovarian cancer precision therapeutics. Front. Pharmacol. 14, 1276209. 10.3389/fphar.2023.1276209 38239204 PMC10794632

[B2] Armstrong D. K. Alvarez R. D. Bakkum-Gamez J. N. Barroilhet L. Behbakht K. Berchuck A. (2021). Ovarian cancer, version 2.2020, NCCN clinical practice guidelines in oncology. J. Natl. Compr. Cancer Netw. 19 (2), 191–226. 10.6004/jnccn.2021.0007 33545690

[B3] Baranello M. P. Bauer L. Benoit D. S. (2014). Poly(styrene-alt-maleic anhydride)-based diblock copolymer micelles exhibit versatile hydrophobic drug loading, drug-dependent release, and internalization by multidrug resistant ovarian cancer cells. Biomacromolecules 15 (7), 2629–2641. 10.1021/bm500468d 24955779

[B4] Barenholz Y. (2012). Doxil®--the first FDA-approved nano-drug: lessons learned. J. Control. Release 160 (2), 117–134. 10.1016/j.jconrel.2012.03.020 22484195

[B5] Bhandari M. Raj S. Kumar A. Kaur D. P. (2022). Bibliometric analysis on exploitation of biogenic gold and silver nanoparticles in breast, ovarian and cervical cancer therapy. Front. Pharmacol. 13, 1035769. 10.3389/fphar.2022.1035769 36618941 PMC9818348

[B6] Bhattacharya S. Anjum M. M. Patel K. K. (2022). Gemcitabine cationic polymeric nanoparticles against ovarian cancer: formulation, characterization, and targeted drug delivery. Drug Deliv. 29 (1), 1060–1074. 10.1080/10717544.2022.2058645 35363113 PMC8979509

[B7] Chen D. Li B. Lei T. Nie M. Yang Y. Congjia X. (2021). Selective mediation of ovarian cancer SKOV3 cells death by pristine carbon quantum dots/Cu2O composite through targeting matrix metalloproteinases, angiogenic cytokines and cytoskeleton. J. Nanobiotechnol. 19 (1), 68. 10.1186/s12951-021-00813-8 PMC7934478 33663548

[B8] Dai X. Li L. Li M. Yan X. Li J. Mao H. (2023). One pot preparation of muti-mode nanoplatform to combat ovarian cancer. Biomed. Pharmacother. 165, 115172. 10.1016/j.biopha.2023.115172 37473681

[B9] Han Q. Li Z. Fu Y. Liu H. Guo H. Guan X. (2023). Analyzing the research landscape: mapping frontiers and hot spots in anti-cancer research using bibliometric analysis and research network pharmacology. Front. Pharmacol. 14, 1256188. 10.3389/fphar.2023.1256188 37745055 PMC10512719

[B10] Henderson E. Huynh G. Wilson K. Plebanski M. Corrie S. (2021). The development of nanoparticles for the detection and imaging of ovarian cancers. Biomedicines 9 (11), 1554. 10.3390/biomedicines9111554 34829783 PMC8615601

[B11] Khan M. A. Vikramdeo K. S. Sudan S. K. Singh S. Wilhite A. Dasgupta S. (2021). Platinum-resistant ovarian cancer: from drug resistance mechanisms to liquid biopsy-based biomarkers for disease management. Semin. Cancer Biol. 77, 99–109. 10.1016/j.semcancer.2021.08.005 34418576 PMC8665066

[B12] Lheureux S. Braunstein M. Oza A. M. (2019). Epithelial ovarian cancer: evolution of management in the era of precision medicine. CA A Cancer J. Clin. 69 (4), 280–304. 10.3322/caac.21559 31099893

[B13] Li Q. Shi Z. Zhang F. Zeng W. Zhu D. Mei L. (2022). Symphony of nanomaterials and immunotherapy based on the cancer-immunity cycle. Acta Pharm. Sin. B 12 (1), 107–134. 10.1016/j.apsb.2021.05.031 35127375 PMC8799879

[B14] Li Y. Gao Y. Zhang X. Guo H. Gao H. (2020). Nanoparticles in precision medicine for ovarian cancer: from chemotherapy to immunotherapy. Int. J. Pharm. 591, 119986. 10.1016/j.ijpharm.2020.119986 33069895

[B15] Liang X. Yang Y. Huang C. Ye Z. Lai W. Luo J. (2023). cRGD-targeted heparin nanoparticles for effective dual drug treatment of cisplatin-resistant ovarian cancer. J. Control. Release. 356, 691–701. 10.1016/j.jconrel.2023.03.017 36933699

[B16] Liu Q. Pu T. Zhou X. Sun J. Yuan W. Zhang S. (2024). A follicle-stimulating hormone receptor-targeted near-infrared fluorescent probe for tumor-selective imaging and photothermal therapy. Mater. Today bio. 24, 100904. 10.1016/j.mtbio.2023.100904 PMC10733693 38130428

[B17] Lustberg M. B. Kuderer N. M. Desai A. Bergerot C. Lyman G. H. (2023). Mitigating long-term and delayed adverse events associated with cancer treatment: implications for survivorship. Nat. Rev. Clin. Oncol. 20 (8), 527–542. 10.1038/s41571-023-00776-9 37231127 PMC10211308

[B18] National Comprehensive Cancer Network (2023). Ovarian cancer including fallopian tube cancer and primary peritoneal cancer. https://www.nccn.org/guidlines.

[B19] Nirmala M. J. Kizhuveetil U. Johnson A. Nagarajan R. Muthuvijayan V. (2023). Cancer nanomedicine: a review of nano-therapeutics and challenges ahead. RSC Adv. 13 (13), 8606–8629. 10.1039/d2ra07863e 36926304 PMC10013677

[B20] Pu T. Liu Y. Pei Y. Peng J. Wang Z. Du M. (2023). NIR-II fluorescence imaging for the detection and resection of cancerous foci and lymph nodes in early-stage orthotopic and advanced-stage metastatic ovarian cancer models. ACS Appl Mater Interfaces. 15 (27), 32226–32239. 10.1021/acsami.3c04949 37385963 PMC10347426

[B21] Qu H. Yang J. Li S. Xu J. Zhou X. Xue X. (2023). Programmed-response cross-linked nanocarrier for multidrug-resistant ovarian cancer treatment. J. Control. Release. 357, 274–286. 10.1016/j.jconrel.2023.03.031 36958401

[B22] Rajitha B. Malla R. R. Vadde R. Kasa P. Prasad G. L. V. Farran B. (2021). Horizons of nanotechnology applications in female specific cancers. Semin. Cancer Biol. 69, 376–390. 10.1016/j.semcancer.2019.07.005 31301361

[B23] Siegel R. L. Miller K. D. Jemal A. (2019). Cancer statistics, 2019. A Cancer J. Clin. 69 (1), 7–34. 10.3322/caac.21551 30620402

[B24] Sung H. Ferlay J. Siegel R. L. Laversanne M. Soerjomataram I. Jemal A. (2021). Global cancer statistics 2020: GLOBOCAN estimates of incidence and mortality worldwide for 36 cancers in 185 countries. CA a cancer J. Clin. 71 (3), 209–249. 10.3322/caac.21660 33538338

[B25] Torre L. A. Trabert B. Desantis C. E. Miller K. D. Samimi G. Runowicz C. D. (2018). Ovarian cancer statistics, 2018. A Cancer J. Clin. 68 (4), 284–296. 10.3322/caac.21456 PMC6621554 29809280

[B26] Williams R. M. Lee C. Galassi T. V. Harvey J. D. Leicher R. Sirenko M. (2018). Noninvasive ovarian cancer biomarker detection via an optical nanosensor implant. Sci. Adv. 4 (4),eaaq1090. 10.1126/sciadv.aaq1090 29675469 PMC5906074

[B27] Zhai J. Luwor R. B. Ahmed N. Escalona R. Tan F. H. Fong C. (2018). Paclitaxel-loaded self-assembled lipid nanoparticles as targeted drug delivery systems for the treatment of aggressive ovarian cancer. Appl Mater Interfaces. 10 (30), 25174–25185. 10.1021/acsami.8b08125 29963859

[B28] Zhang J. Ding H. Zhang F. Xu Y. Liang W. Huang L. (2023). New trends in diagnosing and treating ovarian cancer using nanotechnology. Front. Bioeng. Biotechnol. 11, 1160985. 10.3389/fbioe.2023.1160985 37082219 PMC10110946

[B29] Zhang P. Meng J. Li Y. Yang C. Hou Y. Tang W. (2021). Nanotechnology-enhanced immunotherapy for metastatic cancer. Innovation-Amsterdam 2 (4), 100174. 10.1016/j.xinn.2021.100174 PMC8571799 34766099

[B30] Zhao J. Zou F. Zhu J. Huang C. Bu F. Zhu Z. (2022). Nano-drug delivery system for pancreatic cancer: a visualization and bibliometric analysis. Front. Pharmacol. 13, 1025618. 10.3389/fphar.2022.1025618 36330100 PMC9622975

