# Supplementary material for: Current trends and emerging patterns in the application of nanomaterials for ovarian cancer research: a bibliometric analysis
Source: Front Pharmacol. 2024 Mar 8;15:1344855. doi: 10.3389/fphar.2024.1344855 (PMC10957662; doi:10.3389/fphar.2024.1344855)
Supplement: Supplementary file 1 [file Table1.DOCX]

| Rank | Journal | Record | Country | IF(JCR 2022 ) | JCR  quatile |
| --- | --- | --- | --- | --- | --- |
| 1 | International Journal Of Nanomedicine | 85 | NEW ZEALAND | 8.0 | Q1 |
| 2 | Journal Of Controlled Release | 68 | NETHERLANDS | 10.8 | Q1 |
| 3 | Biomaterials | 56 | NETHERLANDS | 14 | Q1 |
| 4 | Acs Applied Materials & Interfaces | 49 | USA | 9.5 | Q1 |
| 5 | International Journal Of Pharmaceutics | 49 | NETHERLANDS | 5.8 | Q1 |
| 6 | Acs Nano | 45 | USA | 17.1 | Q1 |
| 7 | Molecular Pharmaceutics | 45 | USA | 4.9 | Q1 |
| 8 | Scientific Reports | 38 | ENGLAND | 4.6 | Q1 |
| 9 | Nanomedicine-Nanotechnology Biology And Medicine | 36 | NETHERLANDS | 5.4 | Q2 |
| 10 | Colloids And Surfaces B-Biointerfaces | 34 | NETHERLANDS | 5.8 | Q1 |
| 11 | Journal Of Materials Chemistry B | 32 | ENGLAND | 7.0 | Q1 |
| 12 | Bioconjugate Chemistry | 29 | USA | 4.7 | Q1 |
| 13 | Pharmaceutics | 28 | SWITZERLAND | 5.4 | Q1 |
| 14 | Analytical Chemistry | 24 | USA | 7.4 | Q1 |
| 15 | Biosensors & Bioelectronics | 24 | NETHERLANDS | 12.6 | Q1 |
| 16 | Journal Of Nanobiotechnology | 24 | ENGLAND | 10.2 | Q1 |
| 17 | Pharmaceutical Research | 24 | GERMANY | 3.7 | Q2 |
| 18 | Rsc Advances | 24 | ENGLAND | 3.9 | Q1 |
| 19 | Cancers | 23 | SWITZERLAND | 5.2 | Q2 |
| 20 | Drug Delivery | 23 | USA | 6.0 | Q1 |

TABLE 1 Top 10 journals in terms of the number of published papers.

TABLE 2 Top 10 cited publications

| Rank | Co-cited references | Total Citations | Centrality | Journal | IF(2022 year) | Corresponding  author’s country |
| --- | --- | --- | --- | --- | --- | --- |
| 1 | CA: A Cancer Journal for Clinicians | 152 | 1 | CA-CANCER J CLIN | Q1/  254.7 | USA |
| 2 | Ovarian cancer statistics, 2018 | 44 | 0.08 | CA-CANCER J CLIN | Q1/  254.7 | USA |
| 3 | CA: A Cancer Journal for Clinicians | 32 | 0.25 | CA-CANCER J CLIN | Q1/  254.7 | USA |
| 4 | CA: a cancer journal for clinicians | 32 | 0.02 | CA-CANCER J CLIN | Q1/  254.7 | USA |
| 5 | Cancer nanomedicine: progress, challenges and opportunities | 29 | 0 | NAT REV CANCER | Q1/  78.5 | ENGLAND |
| 6 | CA: A Cancer Journal for Clinicians | 28 | 0.02 | CA-CANCER J CLIN | Q1/  254.7 | USA |
| 7 | Epithelial ovarian cancer: Evolution of management in the era of precision medicine | 27 | 0.76 | CA-CANCER J CLIN | Q1/  286.130 | USA |
| 8 | Analysis of nanoparticle delivery to tumours | 26 | 0 | NAT REV MATER | Q1/  83.5 | ENGLAND |
| 9 | Ovarian Cancer: An Integrated Review | 24 | 0 | SEMIN ONCOL NURS | Q2/  2.2 | USA |
| 10 | Cancer nanotechnology: The impact of passive and active targeting in the era of modern cancer biology | 22 | 0.05 | ADV DRUG DELIVER REV | Q1/  16.1 | NETHERLANDS |
